# Intelligent host engineering for metabolic flux optimisation in biotechnology

**DOI:** 10.1042/BCJ20210535

**Published:** 2021-10-21

**Authors:** Lachlan J. Munro, Douglas B. Kell

**Affiliations:** 1 Novo Nordisk Foundation Centre for Biosustainability, Technical University of Denmark, Building 220, Kemitorvet, 2800 Kgs. Lyngby, Denmark; 2 Department of Biochemistry and Systems Biology, Institute of Systems, Molecular and Integrative Biology, University of Liverpool, Crown St, Liverpool L69 7ZB, U.K.; 3 Mellizyme Biotechnology Ltd, IC1, Liverpool Science Park, 131 Mount Pleasant, Liverpool L3 5TF, U.K.

**Keywords:** flux, host, metabolomics, optimisation, proteomics, transcriptomics

## Abstract

Optimising the function of a protein of length *N* amino acids by directed evolution involves navigating a ‘search space’ of possible sequences of some 20*^N^*. Optimising the expression levels of *P* proteins that materially affect host performance, each of which might also take 20 (logarithmically spaced) values, implies a similar search space of 20*^P^*. In this combinatorial sense, then, the problems of directed protein evolution and of host engineering are broadly equivalent. In practice, however, they have different means for avoiding the inevitable difficulties of implementation. The spare capacity exhibited in metabolic networks implies that host engineering may admit substantial increases in flux to targets of interest. Thus, we rehearse the relevant issues for those wishing to understand and exploit those modern genome-wide host engineering tools and thinking that have been designed and developed to optimise fluxes towards desirable products in biotechnological processes, with a focus on microbial systems. The aim throughput is ‘making such biology predictable’. Strategies have been aimed at both transcription and translation, especially for regulatory processes that can affect multiple targets. However, because there is a limit on how much protein a cell can produce, increasing *k*_cat_ in selected targets may be a better strategy than increasing protein expression levels for optimal host engineering.

## Introduction

Much of microbial biotechnology consists conceptually of two main optimisation problems [1]: (i) deciding which proteins whose levels should be changed, and (ii) by which amounts. The former is ostensibly somewhat simpler, e.g. when a specific enzyme is the target for overproduction, since the assumption is then that the aim is simply the maximal production of the active target (whether intracellularly or in a secreted form). Where the overproduction of a small molecule is the target the optimal levels of individual metabolic network enzyme proteins depend on their specific kinetic properties and the consequent distribution of flux control (e.g. [2–8]). Since both circumstances ultimately seek to maximise the flux to the product of interest, we shall discuss them both, albeit mostly at a high level. Recognising that many pathways are poorly expressed in their natural hosts we shall be somewhat organism-agnostic [9,10], (though we largely ignore cell-free systems) since we are more interested in the principles (whether microscopic [11] or macroscopic [12]) than the minutiae.

The possible number of discrete manipulations one can perform on a given system is referred to as the ‘search space’. The overriding issue is that the number of changes one *might* make scales exponentially with the number of those considered, and is simply astronomical; the trick is to navigate the search space intelligently [13]. Modern methods, especially those recognising the potential of synthetic biology and host engineering to make ‘anything’ (e.g. [14–23]), are improving both computational [24] and experimental approaches. The main means of making such navigation more effective is by seeking to recognise those areas that are most ‘important’ or ‘difficult’ for the problem of interest, and focusing on them; this is generally true of combinatorial search problems (and to illustrate this, a nice example is given by the means by which the Eternity puzzle https://en.wikipedia.org/wiki/Eternity_puzzle was solved).

## Forward and inverse problems, and how the latter are now within range

**Figure 1.**
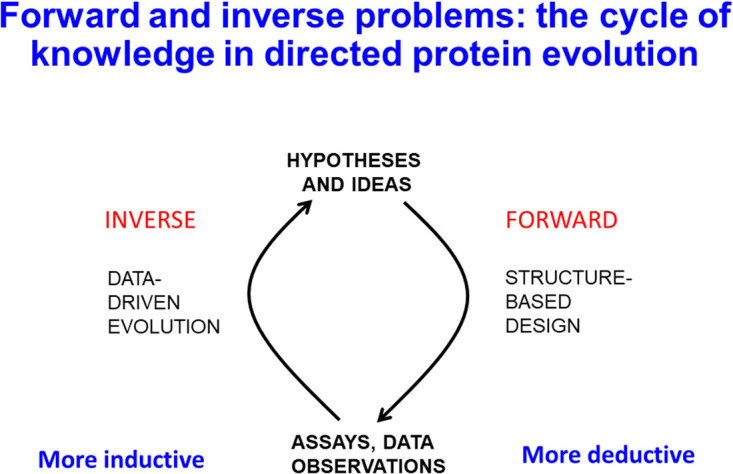
The cycle of knowledge in directed protein evolution. It is useful to contrast the worlds of (i) mental constructs including ideas and hypotheses from (ii) more physical worlds that include data and ‘observations’. Their interrelations are iterative but their nature depends on their directionality. In the post-genomic era, there has rightly been a trend away from the primacy in the biology of hypothesis-dependent deductive reasoning towards data-driven biology in which the best explanations are induced from available data.

We find it useful to classify problems into ‘forward’ and ‘inverse’ problems, because (Figure 1) this is in fact how they are commonly presented [13]. In areas such as drug discovery, a typical forward problem might be represented by starting with a set of structures and paired quantitative properties or activities (QSAR/QSPR) with which one can set up a model or a nonlinear mapping in which the molecular structures are the inputs and the activities are the outputs. A good model will be able to ‘generalise’, in the sense that it can give accurate predictions for novel molecular structure. On a good day (such as here [25]), it will even be able to extrapolate, to make predictions of activities larger than it ever saw when it was being trained. This can be seen as a ‘forward’ problem (‘have molecule, want to predict properties’), nowadays known as a ‘discriminative’ problem [26–28]. However, what we really wish to solve [29,30] is the normally far harder *inverse* problem (‘have desired properties, want molecules’), nowadays referred to as a ‘generative’ problem [26,27,31–35] since the output is (the generation of) the solution of interest. Such generative methods are now well known in the image and natural language processing, and are becoming available in all kinds of related areas of present interest such as drug discovery [36] and protein sequence generation (e.g. [37–40]). Thus, while we have (and can more or less easily create [41]) reasonable whole-genome models of all kinds of microbes (and see below), what we effectively need to solve here again is the ‘inverse problem’ [30,42]. For organism optimisation this is mainly ‘have desired flux, need to optimise the gene sequences and expression profiles of my producer organism to create it’.

## Combinatorial problems of genetic sequences

The genetic search space in biology is enormous; even considering just a 30 mer of the standard nucleic acid bases can produce 4^30^ (∼10^18^) different sequences. The enormity of this number can be illustrated by the fact that if each such sequence was arrayed as a 5 μm spot the array would take up an area of ∼29 km^2^ [43]. Obviously, the number of bases in just a smallish bacterial genome such as that of *Escherichia coli* MG1655 is some 10^5^ times greater than 30, and it remains the case that we still know next to nothing about approximately one-third of the genes encoded therein [44], the so-called y-genes [45]. The expression levels of the identical protein sequence can vary several 100-fold just by changing the codons used [46], mainly because of the expression levels [47,48] and the stability of mRNA [49,50], as well as because of codon bias [51] and for other reasons [52]. Also, note that obtaining the best expression of the active protein is not simply a question of using the commonest codons [53], since (i) over-usage of an individual codon will necessarily deplete its tRNA, and (ii) sometimes it is necessary to *slow down* protein expression so as to avoid inclusion body formation [54]. Consequently, the problem is not made easier by substituting the term ‘nucleic acid bases’ in the above reasoning by the words ‘amino acids’ or ‘codons’. Indeed, the control of gene expression is distributed over the whole genome [55].

## Combinatorial problems of protein engineering

Considering just the 20 ‘common’ amino acids, the number of sequence variants for *M* substitutions in a given protein of *N* amino acids is $\frac{19M.N!}{(N-M)!M!}$ [56]. For a protein of 300 amino acids with random changes in just 1, 2, or 3 amino acids in the whole protein this evaluates to 5700, ca 16 million, and ca 30 billion, while even for a small protein of *N *= 165 amino acids (smaller than half that of the average protein length in Uniprot), the number of variants exceeds 10^15^ when *M* = 8. If we wish to include insertions and deletions, they can be considered as simply increasing the length of *N* and the number of variants to 21 (with a ‘gap’ being coded as a 21st amino acid). Obviously, if we just consider a fixed number of positions *N* the number of possibilities scales as 20*^N^* if any amino acid substitution is allowed. At all events, the dimensionality of the problem is equal to the number of things that can be varied, and it is the exponent in a relationship where the base (in the mathematical sense) is the number of values that it can take. In contrast, for a 165-, 350-, or 700-residue protein, although the number of ways of finding ‘the best’ five amino acids to vary is, respectively ∼10^8^, ∼2.10^10^, and 10^12^, exhaustive search of those five amino acids always involves ‘just’ 20^5^ = 3.2 million variants. Thus strategies (such as ProSAR [57–60]) that seek the best elements to mutate at all, even in the necessary absence of epistatic analyses (see below), have considerable merit.

## Directed protein evolution

**Figure 2.**
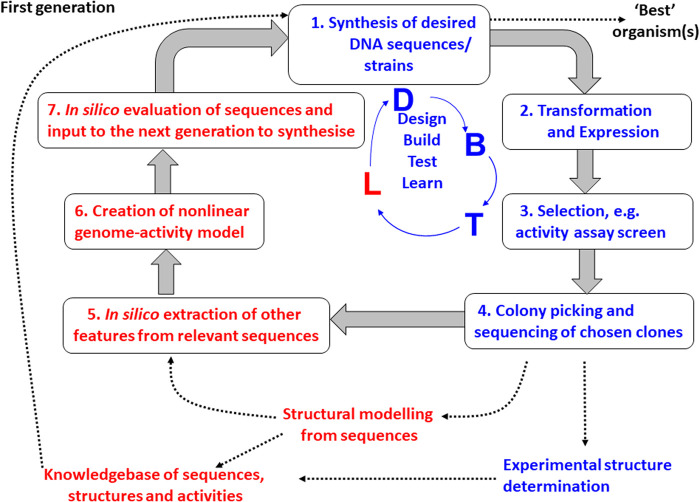
The design–build–test–learn (DBTL) paradigm for engineering biology. Although usually considered solely at the level of protein directed evolution (and on which this diagram is based [13]), the DBTL strategy applies equally to host engineering.

Overall, the solution to such a combinatorial search problem, as is used by the biology of course, is not to try to make these massive numbers of changes ‘in one go’ but to build on earlier successes in a generational or evolutionary manner, known in protein engineering as the design–build–test–learn (DBTL) cycle (Figure 2).

**Figure 3.**
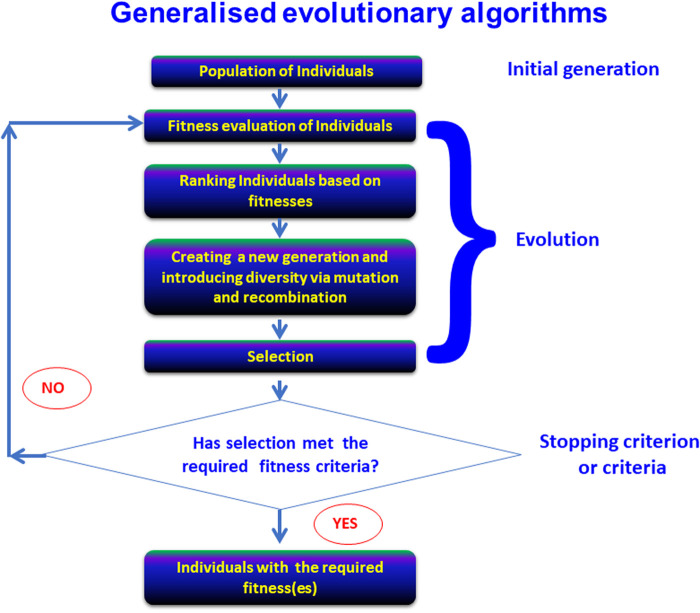
Generalized evolutionary algorithms. The elements of an evolutionary algorithm, in which a population of candidate solutions are mutated and recombined, iteratively with selection, to develop improved variants. Based in part on [95].

Algorithmic strategies for doing so *in general* (including in physical sciences and engineering) are known variously as ‘genetic algorithms’ or ‘evolutionary computing’, and come in a variety of flavours (e.g. [61–69]). They have become well known for individual proteins in the form of directed evolution, as popularised in particular by Frances Arnold (e.g. [70–73]). Some recent reviews include [74–81]. Increasingly, the use of ‘deep mutational scanning’ [82–90], sometimes coupled to FACS-based sorting [91] (‘sort-seq’ [52,82,83,92]), is making available large amounts of sequence-activity pairs [85]. (We ourselves made available one million paired aptamer activity sequences in 2010 [93].) The general structure of an evolutionary algorithm is outlined in Figure 3. As (to some degree [94]) with natural evolution and organism breeding, it is up to the experimenter to select individuals to mutate or to recombine, specifically as one seeks combinations of traits that overall provide the desired phenotype.

## The problems of landscape ruggedness and epistasis

**Figure 4.**
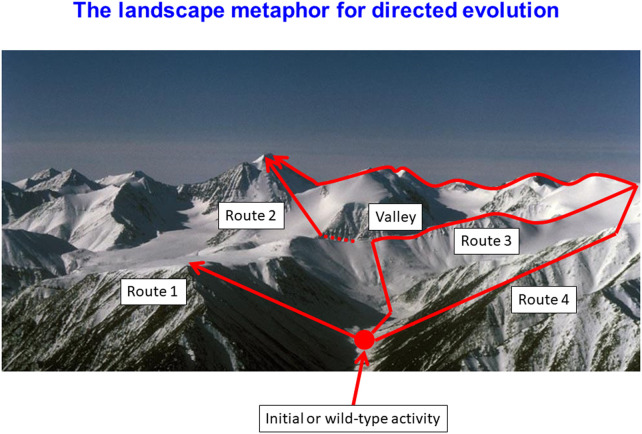
The landscape metaphor for understanding genotype–phenotype relationships in directed evolution and similar protein optimisation experiments. The *X*–*Y* co-ordinates indicate where one is in the sequence space, while the height indicates the value of the desired objective function(s). Reproduced from an open-access publication at [13].

Notwithstanding the numerical combinatorial problems, the biggest problem in natural evolution involves that of epistasis, i.e. the very common circumstances in which the ‘best’ amino acid at a certain location depends on the precise nature of the amino acid at one or more other residues. The commonest way to think of these problems is in terms of the fitness landscape metaphor [96], as illustrated in Figure 4. In this representation, ‘where’ one is in the multidimensional search space is encoded via the *X*- and *Y*- co-ordinates, while the value of the (composite desired) property of interest, or the fitness, is represented as the height. Epistasis manifests as a sort of ruggedness in the landscape, and is more-or-less inevitable when residues that are ‘distant’ in the primary sequence are in contact; indeed their covariance provides an importance strategy for *detecting* such contacts from sequences alone (e.g. [97–100]). In particular, ‘sign’ epistasis occurs when A is better than B at location one when C is at location 2, but B is better than A at location one when D is at location two. It is easy to understand this in simple biophysical terms with respect to the likelihood of contact formation, in this case via ion pairs, if A, B, C, and D are, respectively, glutamate, lysine, arginine and aspartate. Indeed, the covariation of residues in protein families is widely used as a means of predicting 3D structure from sequence alone [97,98,101–103].

The ‘ruggedness’ of the fitness landscape is widely taken to reflect the ease with which it may be searched, and which kinds of search algorithms may be optimal [62,93,104]. Biological landscapes tend to be somewhat rugged, but not pathologically so [105,106]. The so-called NK landscapes (e.g. [107–110]) are convenient models, and are completely non-rugged when *K *= 0; experimentally, we found *K *∼ 1 for protein binding to DNA sequences [93]. Ruggedness necessarily increases as protein length *L* increases, and reasonable routes joining everything upwards or neutrally so as to escape local minima decrease [111], though they do exist in high dimensions [112].

Such sign epistasis is both common and highly important [113–116], and is especially responsible for ruggedness and local isolation under selection. From what we know (e.g. [117]), while pairwise epistasis of this type is indeed very common [118], including in adjacent residues [119], higher-order epistasis is somewhat less so (plausibly for steric reasons). Armed with paired sequence and activity values, all one can do is to seek to interpolate between the few positions with known values and those without. However, if one simply keeps climbing locally one is inevitably likely to be trapped in a local minimum (or maximum in the landscape metaphor) from which it is very hard to escape by mutation alone. Weak mutation and strong selection are commonplace in natural evolution [120–127]) and consequently tend to disfavour lower fitnesses [128], exacerbating the problem of being trapped in a local minimum. This largely constrains natural evolution, and means that we can anticipate great improvements in organisms by seeking previously unknown sequences distant from known ‘peaks’.

Overall, then, the concept of epistasis implies that there is no monotonic ordering of the utility or performance of individual residues within a complete fitness landscape, and that depending on what else is going on there is some kind of a bell-shaped curve relating the utility of a given amino acid in the performance of a protein, to the rest of the protein landscape when that is allowed to be varied. As we shall see, and it is in fact inevitable, this is commonly mirrored more generally.

## Combinatorial problems of host engineering

As presaged, the basic combinatorial problem of host engineering [129] is largely equivalent to that of protein engineering. We have *P* enzymes, each of which might be expressed at *Q* levels (to make life more reasonable in practice we would let these levels vary logarithmically, so 20 levels of a twofold increment gives a range of just over 10^6^ (2^20^ = 1 048 576)). Our problem comes (this is easily checked using the ‘COMBIN’ function in a spreadsheet programme such as MS-Excel, or online) because again the number of combinations NC explodes as *P* increases (for *Q* = 20, NC ∼ 10^14^ for *P* = 50, NC > 10^20^ for *P* = 100, and NC > 10^30^ for *P* = 300, which is lower than one-tenth of the number of gene products in *E. coli*). However, for *P* = 100, NC is only 100 and 4950 when just one or two variant levels are introduced that differ from those of the wild type (WT), respectively. As with any combinatorial search problem, appropriate application of modern Bayesian, machine learning, and design of experiment principles can assist with finding optimal combinations (e.g. [130–133].

## The optimisation of flux in metabolic networks; metabolic control analysis

**Figure 5.**
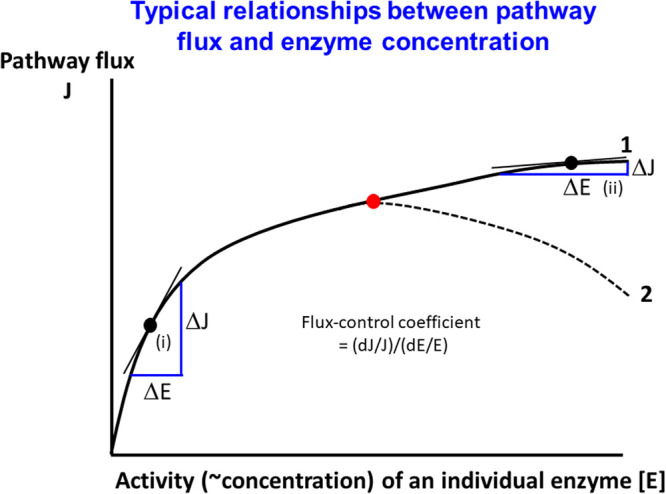
Some potential relationships between the activity of an individual enzyme in a metabolic ‘pathway’ and a flux of interest. For these purposes, we consider that enzyme concentration and activity are proportionate. The broadly expected result is similar to that of curve 1, where there is a monotonic increase in flux as the enzyme's activity is increased. At lower enzyme activities (i) the slope of the tangent is reasonably high, while at higher activities (ii) a further increase in enzyme activity has little effect on pathway flux and the slope is correspondingly low. In some circumstances (curve 2), whether because of pleiotropic effects or because of the negative effects of an increased protein burden (see text), increases in enzyme activity beyond an optimum (marked in red) lead to decreases in pathway flux. The flux-control coefficient is the normalised slope relating pathway flux to enzyme activity at the operating point of interest.

Much evidence exists (modulo ‘bet hedging’ [134–138]), that the majority of organisms in a population seek to maximise their instantaneous growth rate (the flux to biomass) [12,139], and thus understanding the control of flux is a core issue, whatever the flux of interest. The typical relationship between the flux through a metabolic pathway or network and the concentration of an individual enzyme typically follows some kind of curve like a rectangular hyperbola (similar to that relating activity to substrate concentration in the simplest Michaelis–Menten equation). An illustration is given in Figure 5.

Understanding why this is so is the territory of metabolic control analysis (MCA), which originated in the work of Kacser and Burns [140–142] and of Heinrich and Rapoport [143,144]. We refer readers to some reviews (e.g. [2,3,5–8,145–147]) and an online tutorial http://dbkgroup.org/metabolic-control-analysis/. MCA can be seen as a kind of local sensitivity analysis [148] (cf. [149]), in which the sensitivities (known as control coefficients, illustrated for a flux-control coefficient in Figure 5) add up either to zero or to one. The chief points of MCA for our purposes are that (i) every enzyme can contribute to the control of flux, but because their *flux*-control coefficients add up to zero the contribution of individual enzymes is mostly small, (ii) the distribution of control varies as the activity of an individual enzyme is increased (this is somewhat equivalent to epistasis), (iii) because it is activities that matter and because enzyme concentrations cannot be increased without limit, it is better to increase them directly (through increasing individual *k*_cat_ values) rather than by increasing enzyme expression levels (in terms of kinetics this might only differ during transients, not in the steady state [42]), (iv) the best way to increase fluxes is to modulate multiple enzyme activities simultaneously [5,150], (v) because the sum of *concentration*-control coefficients is zero, individual steps can and do have substantial effects on the concentrations of metabolic intermediates (this is precisely why the metabolome can serve to amplify comparatively changes in the transcriptome or the proteome [151–153]). However, normally it is fluxes to the product in which one is interested for biotechnology, and in terms of increasing metabolic fluxes, one has to make choices from a combinatorial space [154], because there are necessarily fairly strong limitations on the total amount of protein that can be made by a given organism.

## The concept of ‘spare capacity’

The fact that most enzymes have small flux-control coefficients (because they must add up to one) necessarily means that they must tend to have ‘spare capacity’; this is simply another way of saying that increasing or lowering their activity has relatively little effect of a pathway flux. Such spare capacity also allows for rapid responses in the face of changes in the environment [155–159]. This spare capacity *of itself* implies that there is plenty of ‘room for manoeuvre’ in host engineering. Indeed, the ‘spare capacity’ has been identified explicitly in a variety of systems [160], for instance in mitochondrial respiration (e.g. [161,162]) and others discussed below. This said, some other respiratory systems are barely able to keep pace with the need to oxidise reducing equivalents that can be produced at high rates (e.g. [12,163–166]). Depending on one's point of view of the desirability of forming the relevant products, a failure of spare capacity in some pathways might also be seen as contributing to so-called overflow metabolism [167], as may be evidenced by ‘metabolic footprinting’ [168,169] or ‘exometabolomics’ [170–173]. Ultimately, of course, the ‘adaptability’ of an organism typically depends on the environments in which it has naturally evolved [174,175].

**Figure 6.**
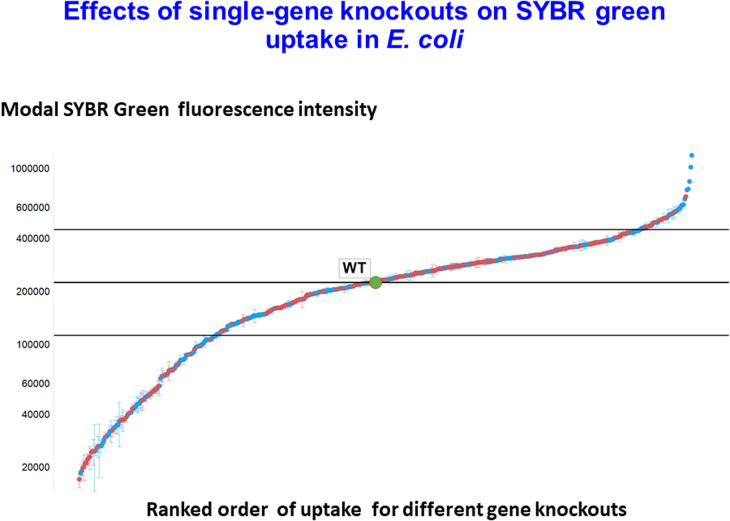
The modal extent of uptake of SYBR Green in single-gene knockouts of *E. coli*. The data reflect flow cytometric analysis of the uptake of the fluorescent/ fluorogenic dye SYBR Green into 530 different single-gene knockouts of *E. coli* from the Keio collection. Data are replotted from the supplementary table given in [179]. Red symbols indicate y-genes (genes of ‘unknown’ function). The wild type (WT) is marked in green. The horizontal lines indicated uptakes of one half or double that of the WT.

Transporters are both massively important for biotechnology (e.g. [176–178]) and variably promiscuous (and see later for transporter engineering). In one recent approach [179,180], we have used flow cytometry to assess the ability of single-gene knockouts of some 530 genes *E. coli* (mostly transporter genes; the full list and datsets are in [179,180]) to take up a variety of fluorescent dyes. Some of the data, for SYBR Green uptake (whose fluorescence is massively enhanced upon binding to DNA), are replotted in Figure 6. The range is some 70-fold, reflecting the ability of multiple transporters to influence the uptake and efflux of the dye. Shown are the value for the WT and lines representing one half of and twofold that uptake (encompassing 361 of the 531 knockouts, ca 68%, studied). As expected, most manipulations have comparatively little effect, but there is a ‘long tail’ [181] (here in either direction) of a few that do. This is quite typical of biology, where it is also worth noting that the flow cytometric analysis of clonal cultures indicates a massive heterogeneity therein, presumably as a result of the differential expression of many hundreds of different enzymes. In some ways, the only surprise is that this variation is so small, and that is likely a result of evolution's necessary selection for robustness (e.g. [182–191]).

## Basic limitations of protein expression: how we cannot just make more of every protein to increase flux

As is well known, microbes adjust their ribosomal content to match their growth rate [12,192,193]. Thus, when the opportunity arises, they can funnel excess amino acids into ribosomal biosynthesis [194]. Indeed, increasing amino acid availability, as in the ‘terrific broth’ [195], does indeed assist protein production. Equally, it has been known for many years that, although there is considerable flexibility [196], cells do, as they must, have limitations on the total amount of protein that they can make [12,197], both as a flux to protein biosynthesis ‘as a whole’ and as a percentage of total biomass. To this extent, then, especially in laboratory cultures in rich media, the ability to biosynthesise protein is essentially a zero-sum game [198,199]: increasing the amount (concentration) of some proteins necessarily means decreasing the concentration of others. Since consensus metabolic networks have become established in standard organisms such as *E. coli* [200] and yeast [201] (and indeed humans [202,203]), attention has thus begun to shift to the wider proteome [204–207]. Experimentally, while the cell may ‘wish’ to retain some spare capacity, sometimes it is simply not possible. Thus, an early study [208] showed that increases in the expression of a variety of glycolytic enzymes in *Zymomonas mobilis* actually *decreased* the glycolytic flux; it was as though the cells were already at an optimal point (as marked in Figure 5 with a red symbol). Other studies [155,209,210] are reviewed by Bruggeman et al. [12]. A recent analysis in baker's yeast [211] by Nielsen and colleagues covers many of the issues. In this work [211], it was found that under nitrogen-limiting conditions, 75% of the total transcriptome and 50% of the proteome were produced in excess of what is necessary to maintain growth. This necessarily implies that there is scope for improving the host for the purposes of the biotechnologist.

## The potential for host proteome optimisation

**Figure 7.**
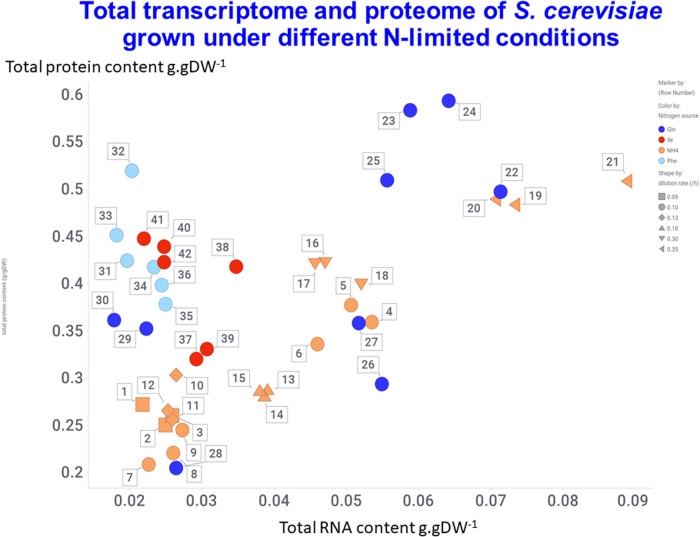
Relationship between absolute proteome and absolute transcriptome in baker's yeast grown under different conditions. Data are replotted from supplementary table S1a of [218]. The dilution rate from 0.1 to 0.35 h^−1^ is encoded by the style of symbol, and the nitrogen source by the colour indicated (optimised for colour-blind legibility via the palettes at http://colorbrewer.org/). Numbers refer to the sample numbers in that table.

Given both the spare capacity, and the fact that many proteins are commonly expressed that are not essential either for cell growth or for assisting fluxes to the product of interest [212,213], it is obvious that attention might usefully be applied to proteome engineering [214–216] as part of host engineering. This becomes especially obvious when it is recognised that the expression levels of even the commonest proteins span some 5 orders of magnitude (in a roughly log-normal distribution) when assessed in baker's yeast using methods that provide absolute numbers [217]. While transcript levels were an important contributor to this variation, the number of protein molecules per transcript in the same study also showed an impressive range, from 40 to 180 000 proteins per transcript [217], implying considerable post-transcriptional control. A more recent study in the same organism detailed absolute protein abundances under some 42 conditions [218], with data replotted therefrom in Figure 7. This serves to show the relatively limited range available in both the total transcriptome and the total proteome in growing cell cultures. Such kinds of datasets (and others such as those in [219] with simple growth as the output) will be of massive value in the future for the purposes of host engineering, as they at once allow one to understand the conditions under which genes are expressed, the strength of the relevant promoters, as well as other features such as genes whose expression varies little and might thus be used for purposes of normalisation [220,221].

## ‘Minimal’ genomes

It is sometimes considered that because biotechnologists often aim to grow cells in rich media, one might usefully delete a lot of the biosynthetic capacity of a cell to ‘streamline’ a genome to make a ‘minimal’ genome. Actually, because of redundancy (A *or* B is required but not both), the number of redundant pairs *n* scales exponentially (as 2*^n^*) so the concept of *the* minimal genome is quite inaccurate. Anyway, any real ‘burden’ comes from the expression, not the possession, of a particular gene, so we focus on strategies that optimise expression. More interesting are essential genes, and a very nice genome-wide study provided a clever method for assessing them [222].

## Transcription vs translation engineering

To vary the amount of a particular protein one can act at the transcriptional or translational levels (or of course both). The former might involve truncations, knockouts, transcription factor engineering (see later), mRNA stability, RNA polymerase engineering, transcription factor binding sites, and direct promoter engineering. A suite of such approaches has been referred to as global transcription machinery engineering (gTME) [223–227]. Translation engineering will tend to have a focus on translation initiation, elongation, and codon optimisation [228,229]. Table 1 gives some examples. We are not aware of any studies that would point experimenters towards a general preference for one or the other (from the MCA analysis above, more likely both), implying that such studies would be of value; it may be, of course, that every problem is more or less bespoke. What is certain, however, is that very little of the search space has ever been covered by natural evolution. A nice example of this is given by the work of Wu et al. [230] who used a transformer model (see [39,231–240]) to assess the ability of various signal peptide sequences (SPs) to induce protein secretion. They found that successful, model-generated SPs were diverse in sequence, sharing as little as 58% sequence identity with the closest known native signal peptide and possessed just 73 ± 9% on average. Unsurprisingly, given the tiny population of sequence space accessed during natural evolution, this is more generally true [241]. Given the many recent advances in deep learning for solving a variety of biological problems (e.g. [31,37–39,242–255]), it is clear that these data-driven [29] strategies will be in the vanguard of the ‘learn’ part of the DBTL cycle.

**Table 1.** Some examples of yield improvement by transcription or translation engineering

Focus	Details/comments	Selected references
*Transcription*		
mRNA stability	Contributes as much to transcription as does codon usage	[46,47]
Promoter engineering	Guided, empirical strategies	[265–268]
	Inducible Trp-T7 for serine production	[269]
	Novel tet-based use of machine learning	[270]
	Random variation on a trc promoter, allowing 60-fold variation in expression levels	[263]
	Promoter library module combinatorics for use in threonine production	[271]
	Reviews	[215,272–279]
	Sigma-factor-specific promoters	[280]
Transcription factor engineering	See below	
DNA/RNA polymerase engineering		[281–284]
Chromosomal integration site	Increased isobutanol production from *E. coli*, involving chromosomal integration at random sites, selection by cell sorting	[285]
	β-carotene synthesis in *Yarrowia lipolytica* by simultaneous integration of a 3-module biosynthetic pathway plus selection by colony colour.	[286]
Riboswitches	Can provide effective control	[287]
σ-factor engineering	Major transcriptional control point	[288]
	Improved antibody production in *E. coli*	[289]
	Extracytoplasmic σ factors	[290]
	Use in cyanobacteria	[291]
Terminator engineering (acts both transcriptionally and translationally)	Increased protein expression through reduced read-through, including Itaconic acid and betaxathin production.	[292–297]
*Translation*		
Codon usage	Strong selection in *S. cerevisiae* leads to plasmid copy variation	[298,299]
	Role in regulating protein folding	[300]
	Review of codon usage tables	[301]
Ribosome binding sites (RBS)	RBS calculator	[302]
	Machine learning in *E. coli*	[303]
	Multiprotein RBS optimisation in various bacteria	[304]
	Review of RBS calculator	[305]
	Phenotypic recording with deep learning, using more than 2.7 M sequence-function pairs	[306]
Translation initiation optimisation	Reviews	[228,307,308]
	Significant increase in serine overproduction	[309]
	5-Methylpyrazine-2-carboxylic acid production	[310]
	2,5-furandicarboxylic acid production	[311]
tRNA engineering	Admits novel codons, of which some can encode non-canonical amino acids	[312]
	tRNA synthetase engineering	[313]

It is generally agreed that while the use of extrachromosomal plasmids is useful for high-throughput screening applications, integration of pathways into the chromosomal DNA of the host organism is ultimately preferable in most production strains due to the well-established instability of plasmids during continuous growth [256,257]. Recently advances in CRISPR and other molecular biology techniques have allowed the integration of reporter genes into a high number of defined genomic sites. Significant variations in expression levels of reporter proteins by the site of genomic integration have been demonstrated in *Saccharomyces* [258], *E. coli* [259,260], *Bacillus subtilis* [261], *Pseudomonas putida* [262], and *Acinetobacter baylyi* [263]. Generally higher levels of expression are seen for integration at sites closer to the origin of replication, as during replication there is in essence a higher copy number of genes that are on the DNA strands replicated first [264]. As discussed in detail above it is rarely a sensible goal to maximise the expression level of all proteins in a relevant pathway, so the genomic integration site of heterologous proteins is an axis on which optimisation can be performed.

## Systems modelling for host engineering

Much of the systems biology agenda (e.g. [30,145,146,314–318]) has recognised that to understand complex, nonlinear systems such as biochemical networks it is wise to model them in parallel with analysing them experimentally. This allows the performance *in silico* of ‘what if?’ kinds of experiments in a manner far less costly than doing them all, allowing one to choose a subset of the most promising. This is also sometimes referred to as e-science [319–321], or having a ‘digital twin’ [322,323] of the process of interest. It is, of course, very well established in fields such as chemical or electronic engineering, where it would be inconceivable to design a process plant or a new chip without modelling it in parallel.

In part, the success of those fields is because we know (because we have designed them) both the wiring diagram of how components or modules interact, and in addition, we know, quantitatively, the input–output characteristics of each module. This allows one to produce what amounts to a series of ordinary differential equations that, given a starting set of conditions, can model the time evolution of the system (by integrating the ordinary differential equations). Such models can be set up in biochemistry-friendly systems such as Copasi [324,325] (http://copasi.org/), CellDesigner [326–329] (http://www.celldesigner.org/), and Cytoscape [330–332] (https://cytoscape.org/). However, prerequisite to this being done accurately is that one has knowledge of the expression levels, kinetic rate equations, and rate constants for each of the steps. This is only rarely achieved (e.g. [333]), even when such details are not known and generalised equations that cover a wide range of force–flux conditions are used [334–336]. Consequently, so-called constraint-based methods have come to the fore. Chief among these is flux balance analysis (FBA) [146,315,317,337–341].

FBA recognises and exploits the massively important ‘stoichiometric’ constraints engendered by the fact that mass must be conserved [342–344], leading to atomic and molecular constraints reflected in reaction stoichiometries, and that consequently only certain kinds of fluxes and flux rations are possible in a known metabolic network. This simple but exceptionally powerful idea, equivalent to Kirchoff's laws in electrical circuit theory, comes into its own when one seeks to *optimise* fluxes to the desired end [345–348] (as in host engineering).

Software for performing FBA is also more or less widely available [340,349], the generic COBRA toolboxes [347,350–353] being especially popular. Such software is much aided by the development of various kinds of linguistic standards for describing systems biology models, such as BioPAX [354–357] (http://www.biopax.org/) and SBML [358] (http://sbml.org/Main_Page).

An especially potent implementation comes from the recognition that if the expression level of a given enzyme is treated as a surrogate for (or an approximation to) the actual flux through that step, then methods that maximise the correlation between predicted and real fluxes, while still admitting mass conservation, can, in fact, predict real fluxes astonishingly well (e.g. [359,360]). Given such a base model, it is then just a question of navigating the space of expression profiles to see those (combinations of) changes that have the greatest effect on the flux of interest. Note, however, that FBA (i) is blind to regulatory effects and (ii) cannot predict metabolite concentrations (only fluxes). Finally, here, it is worth remarking that advanced analyses based on molecular dynamics simulations are beginning to allow the calculation of enzymatic activities and epistatic interactions *de novo* (or at least to account for them) (e.g. [116,361–363]); as with other areas [364–366], the increasing availability of cheap computing will continue to make such methods both more potent and more accurate.

## Genome-wide engineering to improve host performance

As seen in early work in *E. coli* [367], promoter engineering allows one to vary the amount of target enzymes both smoothy and extensively. Of course, nowadays this can be done on a genome-wide scale using methods such as CRISPR–Cas [368,369]. Thus Alper and colleagues [370] assessed the effects of the expression level of all 969 genes that comprise the ‘ito977’ model of *Saccharomyces cerevisiae* metabolism, with overproduction of betaxanthins as one of the objective functions. A particularly important finding was that in a good many cases knockdown rather than complete knockout was preferable, and that there was almost always an optimal level (as per Figure 5) in the range considered. This optimality has been widely reported (e.g. [371–375]), and interestingly (presumably for evolutionary reasons) typically corresponds to the expression level seen in the WT [12]! RNAi engineering can also be used to modulate expression levels [376].

## Transformation engineering

Of the various means of genetic manipulation widely available (transformation, transduction, mating, etc.), transformation by exogenous DNA remains the most popular. This said, transformation using libraries of DNA is far less efficient than one would like [377,378], and it varies considerably with the organism of interest. Some cells [379] such as certain bacilli [380], streptococci [381], acinetobacters [382], and *Vibrio* spp. [383–386] are more-or-less ‘naturally’ competent, which others require considerable optimisation to achieve acceptable rates [387]. A veritable witches’ brew of cocktail components have been considered; at this stage, it seems that an empirical approach is needed for every organism (e.g. [388–393]). There is also the question of whether the vector to be used is intended to be or remain episomal or to integrate by recombination into the host chromosome. These are areas that will require especial attention for improved host engineering.

## CRISPR–Cas-based genome engineering

The arrival of CRISPR–Cas9 and related genome editing tools [394,395] is well enough known as not to need detailed review (and many are available, e.g. [369,396–410]).

A recent advance incorporates the ability to incorporate a simple (barcoded) coupling between the gRNA that might have had an effect and its nature as encoded via a barcode. This is the CRISPR-enabled trackable genome engineering (CREATE) technology developed by Gill and colleagues (e.g. [396,404,411–413]). CREATE uses array-based oligos to synthesise and clone 100s of 1000s of cassettes containing a genome-targeting gRNA covalently linked to a dsDNA repair cassette encoding a designed mutation. After CRISPR/Cas9 genome editing, the frequency of each designed mutant can be tracked by high-throughput sequencing using the CREATE plasmid as a barcode. (A commercial version of this approach is now available as the Onyx™ instrument (https://www.inscripta.com/technology).)

A biotechnological example of the CREATE technology is that for lysine production (a mature, multi-billion $US market [414]) in *E. coli* [412]. Here the authors [412] designed over 16 000 mutations to perturb lysine production, and mapped their contributions toward resistance to a lysine antimetabolite (toxic amino acid analogue). They thereby identified a variety of different routes that can alter pathway function and flux, uncovering mechanisms that would have been difficult to design rationally — many were, in fact, unknown! In the event, mutations in genes linked to transport, biosynthesis, regulation, and degradation were uncovered, with some being as expected (showing the virtue of the strategy) and others—especially in DapF acting as a regulator—being entirely novel. Overall, this strategy provides an exceptionally potent, efficient and effective approach to the principled discovery of ‘novel’ genes involved in any bioprocess of interest that can be run at different ‘levels’ or in different ‘states’.

## Transcription factor engineering

It is a curious fact that much of the community that studies plants has focused on the control of flux via transcription factors (TFs, e.g. for pigment production [415–418]), while microbiologists have tended historically to focus more directly on metabolic networks *per se*. This is starting to change.

The transcription factor-based regulatory network of *E. coli* is probably the best studied (e.g. [419–421], with over 200 TFs [422–424] organised into some 150 regulons [423,425,426]. Independent components analysis (ICA) is a useful, convenient, multivariate linear, and well-established technique for separating mixed signals into orthogonal contributions; it has been used to group these differential gene expression changes into over 300 iModulons [427,428]. One may suppose that semi-supervised methods of deep learning [31] will prove even more rewarding in terms of understanding coregulation.

A related study in yeast manipulated some 47 TFs (via a library containing over 83 000 mutations) affecting over 3000 genes, leading to a substantial improvement in both isopropanol and *n*-butanol tolerance. An analysis of the relevant gene expression changes showed that genes related to glycolysis played a role in the tolerance to isobutanol, while changes in mitochondrial respiration and oxidative phosphorylation were significant for tolerance to both isobutanol and isopropanol.

The number and nature of the genes regulated by TFs can vary considerably, and in a nice strategy Lastiri-Pancardo et al. [429] worked out those whose removal would provide maximal flexibility for the reorganisation of allocation of the rest of the proteome. For instance, feast/famine regulatory proteins/transcription factors [430,431] are common to both archaea and eubacteria; Lrp, in particular, is especially responsive to the concentration of leucine as an indicator of the cell's nutritional status. Overall, TFs seem a particularly useful target for intelligent host engineering (e.g. [432–434], including in biosensors [435–444]).

**Figure 8.**
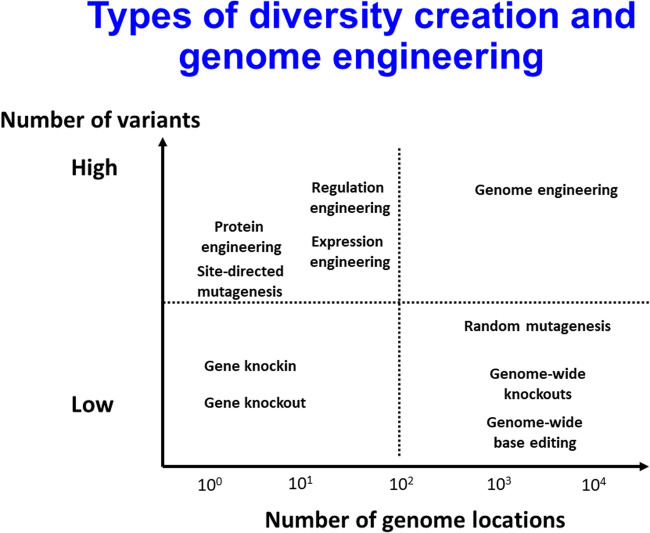
Types of diversity creation and genome engineering. Different strategies for creating strain diversity as part of host engineering, set out as a ‘Boston matrix’ reflecting the variation between difference strategies in terms of the number of variants created and the number of genomic locations tested. Based on the material at https://www.youtube.com/watch?v=tb97SghfL_8&t=256s.

In addition to changing the expression levels of target genes, we will also wish to change their activities, and one obvious means is via mutation. The kinds of diversity creation that can effect mutation are summarised in Figure 8.

## Methods for genome-wide introduction of mutations

Many of the genetic variations that improve the performance of microbial cell factories are not currently possible to design rationally, despite the large degree of genetic knowledge around many platform strains [378]. This is in large part due to the high degree of epistasis and the combinatorial problems discussed in detail above. While advances in AI are rapidly changing this (see above), improvements in microbial cell factories are presently still in many cases being found by wet laboratory techniques (Table 2) that introduce more-or-less random mutations across the genome and then select for strains with desired properties. These strains can be used directly, or with the plummeting costs of next-generation sequencing, beneficial mutations can be identified revealing new mechanisms and targets for further rational design. A further advantage of random mutagenesis relevant to some applications is that strains generated through random mutagenesis are considered ‘GMO free’, which allows one to avoid legal regulations that have been set up around some kinds of so-called genetically modified organisms [445,446].

**Table 2.** Example applications of techniques to introduce genome-wide mutations

Technique	Species	Purpose	Notes	References
UV	*Kluyveromyces marxianus*	Improved ethanol production	Used an automated platform incorporating UV mutagenesis.	[480]
	*Yarrowia lipolytica*	Improved oil production		[481]
Chemical mutagenesis	*Chlorella vulgaris*	Light tolerance		[482]
	*Brettanomyces bruxellensis*	Reduced production of 4-ethylphenol, an undesirable by-product in wine fermentation		[483]
	*Yarrowia lipolytica*	Increased lipid production		[484]
	*Lipomyces starkeyi*	Increased production of triacylglycerol		[485]
Atmospheric and room temperature Plasma mutagenesis	*Zymomonas mobilis*	Acetic acid tolerance		[486]
	*Spirulina platensis*	Astaxanthin production		[487]
	*Escherichia coli*	L-lysine production	Incorporated a biosensor for cell sorting	[488]
	*Actinosynnema pretiosum*	Production of the antibiotic Ansamitocin	Used in combination with genome shuffling	[489]
	*Streptomyces mobaraensis*	Production of the enzyme transglutaminase		
epWGA	*S. cerevisiae*	Ethanol tolerance		[451]
	*Lactobacillus pentosus*	Lactic acid production		[490]
	*Zymomonas mobilis*	Furfural tolerance		[491]
	*E. coli*	Butanol tolerance.		[492]
Serialised ALE	*Saccharomyces cerevisiae*	β-caryophyllene production		[493]
	*Corynebacterium glutamicum*	Glutarate production		[494]
	*E. coli*	Ionic liquid tolerance		[454]
Continuous ALE	*Methylobacterium extorquens*	Methanol tolerance		[495]
	*E. coli*	Conversion to generate all its biomass from CO_2_		[496]
GREACE	*E. coli*	Lysine production		[478]
	*E. coli*	Butanol tolerance		[476]
	*E. coli*	Cadmium resistance		[497]
	*S. cerevisiae*	Acetic acid tolerance, reduced acetaldehyde production		[479]

## UV and chemical mutagenesis

The ability of UV radiation [447] and certain chemicals [448] to cause mutation has been established since the 1930s and 1940s, respectively. While there have been massive advances in the tools available for metabolic engineering and strain generation in the subsequent decades (some of which are outlined below), several recent papers illustrate that there is still utility in using UV radiation and mutagenic chemicals to introduce genetic diversity. These techniques are especially relevant when working with novel or poorly characterised strains for which other tools to introduce variation are lacking, since UV and chemical mutagens cause mutations efficiently in nearly all species.

## Atmospheric and room temperature plasma mutagenesis

Atmospheric and room temperature plasma mutagenesis (ARTM) is a novel technique for introducing random mutagenesis. The application of plasma as a mutagenic agent was first described by Li and colleagues in a 2012 paper [449], in which it was used to generate a mutant library of *Methylosinus trichosporium.* In ARTM, a jet of helium, ionised by an electric field, is blown onto a sample, which (through a yet to be fully elucidated mechanism) causes DNA damage and mutations.

The ARTM technique has, according to a recent review [445], been applied to industrially relevant improvements in over 20 species including both Gram-positive and -negative bacteria, filamentous fungi, yeasts, algae, and cyanobacteria [445]. It has been shown in the *umu* test on *Salmonella typhimurium* that ARTM generates a higher rate of surviving mutated cells than do UV and chemical mutagenesis methods [450]. Despite the apparent advantages, the commercial unit is thus far only available in China, and the publications using ARTM appear to be exclusively from Chinese institutions.

## Error-prone whole-genome amplification

Another technique to introduce mutations across the genome is error-prone whole-genome amplification (epWGA). In this, genomic DNA from the strain of interest is extracted and subjected to error-prone PCR, then retransformed into the initial strain [451]. The transformed cells are subjected to relevant selective pressure, for instance, to isolate strains that have improved property such as a tolerance to an inhibitor or increased product titre. This process can be performed iteratively, and with full genome sequencing beneficial mutations can be identified and isolated to quantify their effects.

## Serialised adaptive laboratory evolution

One of the most widely used and well-established techniques to introduce (i.e. select) beneficial mutations is adaptive laboratory evolution (ALE) [452,453]. ALE is in principle a very simple technique in which cells are cultured under some form of selective pressure, such as the presence of a toxic substance. Cultures are generally serially propagated into media with incremental increases in selective pressure. During this, mutations that confer a fitness advantage accumulate and become fixed in the population. These mutations can then be discovered by sequencing and reintroduced explicitly into a strain of interest, or the evolved strain can be used directly as a platform in downstream applications (e.g. [454,455]).

**Figure 9.**
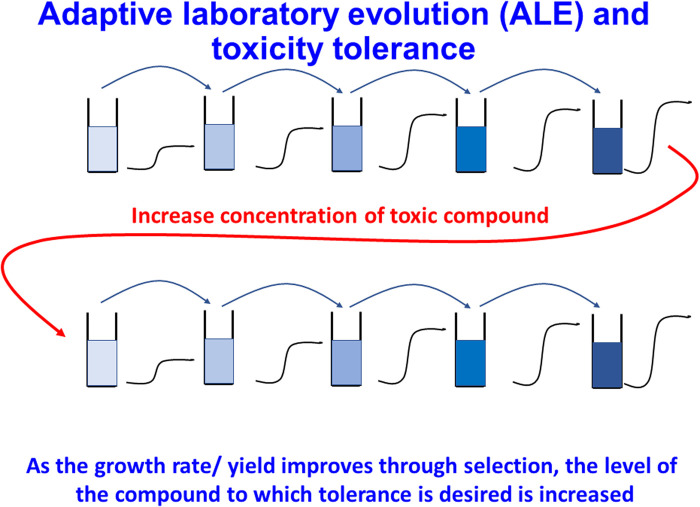
Adaptive laboratory evolution (ALE), illustrated here for tolerance engineering. Cultures are grown in batch mode under conditions in which a stress leads their overall growth rate or yield to be suboptimal. As mutants that are more tolerant to the stress emerge they are selected for and take over the culture, with concomitant increases in growth rate or yield. The magnitude of the stress can then be increased and the process repeated as often as desired.

In the most straightforward use case, tolerance ALE (TALE), cells are propagated in increasing concentrations of some compound that normally inhibits growth in order to improve tolerance (‘tolerance engineering’, Figure 9). Tolerance to toxic environments is still a major limiting factor in achievable yields from microbial cell factories [456,457]. This may be for example toxicity of the desired product (as is the case in fermentative butanol production [458]) or toxic inhibitors present in feed stocks (which is a major challenge in attempts to process lignocellulose hydrolysates [459–461]. ALE may also be used to improve utilisation of a preferred energy source, or to increase product titre directly (although the latter usually requires more advanced experimental design in order to couple production to a fitness advantage [462,463]). A very extensive recent review covering the applications of ALE in more detail is found in [464].

## Continuous adaptive laboratory evolution

While ALE in its simplest form involves serial propagation of cells, continuous evolution techniques utilise variations on what are commonly referred to as ostat bioreactors. These use some form of detection from a growth chamber (commonly OD but such x-stats may also detect pH, dissolved oxygen and many other parameters). Cultures are maintained in a constant state of growth under steady conditions by dilution through automated addition of fresh media along with other supplements or inhibitors. In this way, a constant growth rate and smooth evolution curve can be achieved, compared with the more ‘punctuated equilibrium’ that is the hallmark of serialised ALE [465]. Traditionally cost has been something of a barrier in the use of turbidostats (albeit far from insurmountable [198,199,466–468]), which unlike serialised ALE require specialised detection probes and feedback systems [469]. Recently, however, several open source and low-cost chemostats have become available, reducing the financial barriers to entry at the cost of a requirement for significantly greater hands-on expertise [470–472].

## Genome replication engineering assisted continuous evolution

The adaptive mutations that appear in both serialised and continuous ALE occur through the natural mutations occurring during DNA replication in growing cell populations. Although DNA replication in microbes is generally of very high fidelity (estimated to be on the order of 10^−10^ errors per base pair per generation [473] in wild-type strains), the high density of cells during cultivation (10^8^–10^10^ per ml) still means that enough mutations will occur to generate strains with a fitness advantage. A higher mutation rate may be desirable, however [474], to increase the rate of adaptation or to allow adaptations towards more specialised phenotypes. Indeed, the mutation rate is itself adaptive [475].

The mutation rate in *E. coli* has been increased in a principled way through a technique called genome replication engineering assisted continuous evolution (GREACE) [476–478]. In this approach, a plasmid carrying a modified DNA proofreading element (the *dnaQ* gene) is transformed into the initial strain of interest, and then the transformed cells are subject to continuous ALE. Cells carrying the modified PE plasmid have deficiencies in proofreading ability and, therefore, accumulate mutations at a higher rate than do untransformed cells. Under strong selective pressure, higher mutation rates themselves confer a fitness advantage and the cells carrying the plasmid outcompete those that lose the plasmid. As the deficient proofreading machinery is present on a plasmid as opposed to the genome, once this is removed a strain with the accumulated mutations but a native DNA proofreading system can be recovered, allowing direct use in downstream industrial applications.

Since the initial demonstration of GREACE to generate tolerance to butanol [476], the GREACE methodology has also been extended to *S. cerevisiae*, substituting the *dnaQ* gene with an error-prone DNA polymerase from *S. cerevisiae*. Here it was successfully used to increase the tolerance to acetic acid and reduce the production of acetaldehyde in an ethanol-producing strain [479].

## *V*_max_ vs *k*_cat_

The activity of an enzyme, as expressed in the term *V*_max_, is the product of two terms, viz. the concentration of the enzyme E and its catalytic turnover rate *k*_cat_. Consequently, there are, broadly, two ways to speed up an individual step in a metabolic network: (i) increase the amount of catalyst (*V*_max_) or increase the activity of each catalyst molecule (*k*_cat_). While the former is the more common via well-established promoter engineering methods, we have long taken the view that the latter should be more effective. The reason is simple, i.e. to increase an enzyme concentration 10-fold requires the production of 10-fold more protein, and this is not always possible (see above). Indeed, especially for membrane proteins, the available real estate may be especially limited [498,499]. In contrast, an increase in *k*_cat_ of a 100-fold, which is often easily obtainable in directed evolution programmes, means that one could increase the rate of an individual step by 10-fold while using even ten times *less* of the relevant protein. One example where massive overexpression of a target protein has been used in the overexpression of the efflux transporter for serine [499].

## Membrane transporter engineering

Although we are aiming not to focus excessively on specific areas, we mention transporter engineering because (i) transporters normally exhibit considerable flux control for both substrate influx and product efflux, and (ii) they illustrate more generally how an often-neglected scientific area may benefit from the significant study [500]. In addition, it is (somewhat astonishingly [501]) widely still believed (or at least assumed) that all kinds of substrates simply cross biological membrane via passage through any bilayer that may be present. The facts are otherwise [176,502–509]. Those references rehearse the fact that even tiny molecules like water [510,511] do not pass unhindered through phospholipid bilayers in real biological membranes (whose protein : lipid ratio by mass is often 3 : 1), but require transporters. Recent examples of transporter engineering for biotechnological purposes include glycolipid surfactants [512] and fatty acids [513]. Flow cytometry can provide a convenient means of assessing the activities of certain transporters [179,180].

## Molecular breeding

Classically, the predominance of diploidy in organisms such as penicillia has been seen as a significant disadvantage, as it prevents the emergence of traits that rely on similar activities in both genomes for expression. Indeed, MCA serves to explain the molecular basis of genetic dominance [141], and the use of haploid cells can provide a much great signal : noise in genetic competition experiments [199,514–521]. Yeasts such as those of the genus *Saccharomyces* are of special interest here, since they can sporulate as haploid forms of different mating types that can then interbreed, including interspecifically [522]. Perhaps surprisingly, the effects of this on transcription can be quite modest [523].

## Growth rate engineering

As noted above, natural evolution tends to select for growth rate rather than growth yield [139]. However, typically if the product is not directly growth-associated (e.g. as with ‘secondary’ metabolites in idiophase [10,524]) or with two-stage fed-batch regimes where a growth phase is followed by a production phase, one is wanting cells *not* to grow at the expense of making product [525,526]. Certainly, ‘dormant’ (non-replicating) cells can be quite active metabolically [527–530]. Consequently, although not usually a focus of biotechnology, it remains the case that the more time cells spend in a fermentor non-productively the less good the process. This has led to the consideration of hosts such as *Vibrio natriegens* [50,531–541], whose optimal doubling time can be as little as 7 min, some threefold quicker than the widely quoted 20 min for *E. coli* in rich media. Whether or not organisms such as *V. natriegens* turn out to be valuable production hosts, there is no doubt that understanding how to make cell growth quicker might help enhance the rates of recombinant protein production. Turbidostats [542,543] could be seen as a ‘revved-up’ version of ALE in that they too select for (and demonstrate the levels of any) growth rate enhancement. However, they remain a surprisingly under-utilized system for manipulating microbial physiology, despite many advantages [466,467,544]. Continuing the theme of comparative ‘growth-omics’, the growth rates of yeasts are significantly slower than those of bacteria, the record (in terms of rate of biomass doubling) apparently being *Kluyveromyces marxianus* with a doubling time of some 52 min [498] (a growth rate approximately twice that of *S. cerevisiae* [541,545–547]). This was achieved [498] via a different kind of growth rate selection in a kind of ‘turbidostat’ called a pHauxostat [548–550]).

Consequently, selection for faster growth rates and the concomitant analysis of gene expression changes [546,551–555] would seem to be a powerful means of understanding how to improve cellular performance.

## Medium optimisation and engineering

At one level, the fact that growth rates [219] and expression profiles [198,556–558] differ as different enzymes are expressed at different levels in different growth media is trivial, and essentially describes the whole of microbial physiology. At another level, it is far from trivial because medium optimisation represents yet another combinatorial search problem [559]. If the optimal concentration is considered to be within a known two orders of magnitude and to sit adequately therein within a twofold concentration range, each constituent could take at least 6 values (simply because 100 lies between 2^6^ and 2^7^). With 20 medium constituents, there is then a ‘search space’ of some 6^20^ (∼4.10^15^) recipes to find the optimum. Even just taking metal ions, and noting that approximately half of all enzymes are metalloenzymes [560–562], it is clear that organisms have significant preferences for particular levels of metal ions [563]. In an early example, Weuster-Botz and Wandrey [564] used a genetic algorithm to increase the productivity of formate dehydrogenase in an established fermentation by more than 50%, finding that Ca^++^, Mn^++^, Zn^++^, Cu^++^, and Co^++^ had all been used at excessive levels previously. Obviously, the optimum can also change with the host genotype, so is not fixed even for a given species. Consequently, we feel that automated medium optimisation algorithms should also be at the heart of any host engineering programme. As a classical combinatorial optimisation problem [1,95], this is arguably best attacked by evolutionary algorithms (e.g. [61,62,565–567]); Link and Weuster-Botz [559] give an excellent summary of their applications in medium optimisation, including the rather infrequent cases (e.g. [568]) in which multiple objectives are to be optimised.

## Looking to the future

The variation in expression of individual proteins, even within a nominally homogeneous or axenic culture of an isogenic organism, can vary considerably, leave alone those explicitly differentiated (e.g. [569–572]). This is also becoming ever clearer in differentiated organism via the emerging cell map projects (e.g. [573]) As single-cell transcriptomics, proteomics, and metabolomics become possible, and individual cells are easily sorted in a fluorescence-activated cell sorter, one can contemplate studies in which the expression profiles even of large numbers of nominally isogenic cells are compared with their productivity simultaneously. This may even include understanding of the spatial distribution of proteins within individual cells [574]. One can also imagine a far greater use of the methods of chemical genomics in affecting and understanding cellular behaviour; in this regard, the strategy of chemically induced selective protein degradation [575–580] seems likely to be of significant value.

We have purposely avoided focus on the production of any specific target molecules, since our aim is to help develop the BioEconomy generally. This said, the growth of ‘AI’ and deep learning alluded to above has already shown profound benefits in identifying chemical (e.g. [581–585]) and biosynthetic pathways (e.g. [586,587]), while our own work has developed deep learning methods for molecular generation [588] and molecular similarity [589], for navigating chemical space in a principled way [238], and in particular for predicting the structure of small molecules from their high-resolution mass spectra [239]. In this latter work, we developed a deep neural network with some 400 million interconnections [239], a number that just 3 years ago (writing in July 2021) would have been the largest published. Such has been the growth of large networks (approaching 1% of the interconnections in the human brain) that that number is now too low by a factor of more than 1000-fold [234], necessitating the development of specialist hardware and software to deal with it. Innovations in such kinds of computer engineering, including e.g. in optical computing, will be of considerable benefit. With these large networks has come the question of interpreting precisely how they are doing what they do so well (so-called ‘explainable AI’ or XAI [590–594]). XAI will of necessity lead both to better understanding and to sparser networks, and is an important part of the automation [595–598] (not covered here) that will help to speed up the DBTL cycle enormously.

Classically, electronic circuits were and are predictable because the input/output characteristics of the components are known, and because their wiring diagrams are expertly and precisely controlled by their designers. None of these facts is presently true of biology [599,600], and much of the future in both ‘pure’ organismal bioscience and in biotechnology will thus be about ‘making biology predictable’ [30].

## Concluding remarks

This has been a purposely high-level overview of some of the possibilities in host engineering predicated on genome-wide analyses. Our main aim has been to draw attention to these developments, and to some of the means by which readers who are only loosely acquainted with them can incorporate these methods into their own work.

Take-home messages include

- Host engineering, like directed protein evolution, is a combinatorial search problem.
- Every enzyme potentially has an optimal expression level for every process.
- This is not normally its maximal level, since the maximum amount of protein a cell can produce is fixed, including for a given growth rate; protein synthesis is largely a zero-sum game.
- Changes in the individual concentrations of most enzymes at their operating point necessarily have little effect on fluxes.
- Some areas of transcription and translation effect a more global control and thus can have greater effects and hence serve as better targets for host engineering.
- *k*_cat_ is a much better target for host and protein engineering than is *V*_max._
- Modern methods of modelling, including deep learning, are beginning to provide the ability to assess desirable changes *in silico*, as a prelude to developing a fully predictive biology.

The success of these messages will be judged by the rapidity with which the strategies they contain are adopted.

## Open Access

Open access for this article was enabled by the participation of University of Liverpool in an all-inclusive *Read & Publish* pilot with Portland Press and the Biochemical Society under a transformative agreement with JISC.

## Abbreviations

ALE: adaptive laboratory evolution
ARTM: atmospheric and room temperature plasma mutagenesis
DBTL: design–build–test–learn
epWGA: error-prone whole-genome amplification
FBA: flux balance analysis
MCA: metabolic control analysis
SPs: signal peptide sequences
TFs: transcription factors
WT: wild type
